# Variability of slot size in orthodontic brackets

**DOI:** 10.1002/cre2.219

**Published:** 2019-07-11

**Authors:** Clémentine Lefebvre, Hassan Saadaoui, Jean‐Marc Olive, Stéphane Renaudin, Fabienne Jordana

**Affiliations:** ^1^ Dental Faculty University of Nantes Nantes France; ^2^ Paul Pascal Research Center (CRPP) CNRS (UPR 8641), University of Bordeaux Pessac France; ^3^ I2M Institute of Mechanical Engineering ‐ UMR 5295 CNRS, University of Bordeaux Talence France

**Keywords:** bracket, bracket slot tolerance, slot geometry, slot size

## Abstract

**Objective:**

The accuracy of the information incorporated into brackets is a determining factor for the efficacy of torque applied to teeth. The aim of this study was to compare the dimensions of a bracket's slots with the nominal values announced by the manufacturer.

**Materials and methods:**

A total of 730 maxillary right central brackets manufactured by seven companies (Dentsply Gac, American Orthodontics, Rocky Mountain Orthodontics, GC Orthodontics, 3M Unitek, and Dentaurum) were studied. The sample included 0.018 × 0.025 and 0.022 × 0.028 in., metal and ceramic, conventional and self‐ligating brackets. Images were obtained with an Olympus BX51 optical microscope. Slot dimensions were measured at the base and at the face on both mesial and distal sides using ImageJ software. Data were analyzed using Wilcoxon, sign tests, two‐ and three‐way ANOVA, and Tukey's tests. Intraclass correlation coefficient was employed to assess the intraobserver and interobserver variability. The threshold for statistical significance was *p* ≤ .05.

**Results:**

Statistical analysis showed that the slot dimensions of 90% to 97% of studied brackets were significantly different from nominal values. In general, slot size was oversized, with a face size larger than the base size. Comparison between mesial and distal sides showed that up to 45% of the brackets were significantly asymmetrical. The manufacturer had a significant effect for base and face widths (*p* = .0001) and for length (*p* = .003).

**Conclusion:**

This study shows that a large proportion of measured brackets displays dimensional inaccuracies when compared with stated values. Clinically, the slot oversize and the divergence of slot walls cause an increase of wire‐slot play, inducing a loss of torque control. Practitioners cannot fully trust the precision of used appliances and should be aware that adjustments could be needed in the finishing stages of the treatment.

## 1 INTRODUCTION

The use of straight‐wire techniques implies the insertion of successive straight archwires of increasing cross‐sections, ending with a full‐size arch. The last archwire is expected to fill entirely the bracket slot, allowing then a complete expression of the brackets information.

Contrary to those expectations, the bracket/wire play was proved not to correspond to the theoretical values, showing a systematical increase (Archambault et al., 2009; Arreghini, Lombardo, Mollica, & Siciliani, 2014; Dalstra, Eriksen, Bergamini, & Melsen, 2015). This excess of play is multifactorial. Among these factors, we can point the mode of ligation, the type of material composing the bracket, and the manufacturing imprecision.

Regarding the mode of ligation, studies agree about the superiority of metallic ligation as opposite to elastomeric one in conventional brackets (Dalstra et al., 2015; Fakir et al., 2014; Gioka & Eliades, 2004; Hirai et al., 2012). On the opposite, the actual superiority of active or passive self‐ligating brackets on conventional brackets is not consensual (Badawi, Toogood, Carey, Heo, & Major, 2008; Brauchli, Steineck, & Wichelhaus, 2011; Dalstra et al., 2015; Fleming & Johal, 2010; Katsikogianni, Reimann, Weber, Karp, & Bourauel, 2015; Pandis, Strigou, & Eliades, 2006; Sifakakis, Pandis, Makou, Eliades, & Bourauel, 2010).

A comparison between metal, ceramic, and plastic brackets has shown that slot wall rigidity varied according to the material. Low rigidity brackets such as plastic, and to a lesser degree, metallic ones, allowed more play for the wire than rigid ceramic brackets (Harzer, Bourauel, & Gmyrek, 2004; Matsui, Umezaki, Komazawa, Otsuka, & Suda, 2015; Möller, Klocke, Sadat‐Khonsari, Schlegel, & Kahl‐Nieke, 2009; Morina, Eliades, Pandis, Jäger, & Bourauel, 2008).

The last but not least of the factors causing an increase of play consists in the structural inaccuracies of brackets and archwires, which seem to be due to manufacturing processes. Gioka and Eliades reported in their systematic review that slot surfaces showed microstructural defects and striations (Gioka & Eliades, 2004). Those irregularities appear to be caused by milling processes, and the rough surface generated would prevent the wire from being fully inserted in the bracket slot. Such an obstacle could also be caused by molding processes, as they expose the bracket to shrinkage, but also to a bevel of the slot corners (Major, Carey, Nobes, & Major, 2010). In order to avoid the lack of insertion of the wire into the slot, manufacturers appear to have taken measures such as enlarging the slots and slimming the archwires. It was also shown that a lack of slot wall parallelism could be added to this loss of dimensional accuracy, thus aggravating the bracket/wire play (Cash, Good, Curtis, & McDonald, 2004).

This study had the aim of studying the dimensional precision of a wide sample of marketed bracket series in regards to slot width and length, parallelism of walls, and symmetry between mesial and distal sides.

## 2 MATERIALS AND METHODS

A total of 730 maxillary right central brackets were provided by seven companies (Dentsply GAC, Bohemia, NY; American Orthodontics, Sheboygan, WI; Ormco, Glendora, CA; Rocky Mountain Orthodontics, Denver, CO; GC Orthodontics, Breckerfeld, Germany; 3M Unitek, Monrovia, CA; and Dentaurum, Ispringen, Germany). All brackets were preinformed, 0.018 × 0.025 or 0.022 × 0.028 in., metal or ceramic, conventional or self‐ligating. Self‐ligating bracket clips were either passive or active. In accordance with these criteria, 73 different series (with the same batch) of brackets were put together, and a sample of 10 brackets of each series was randomly selected.

Bracket slot images were obtained using a calibrated Olympus BX51 optical microscope.

**Figure 1:**
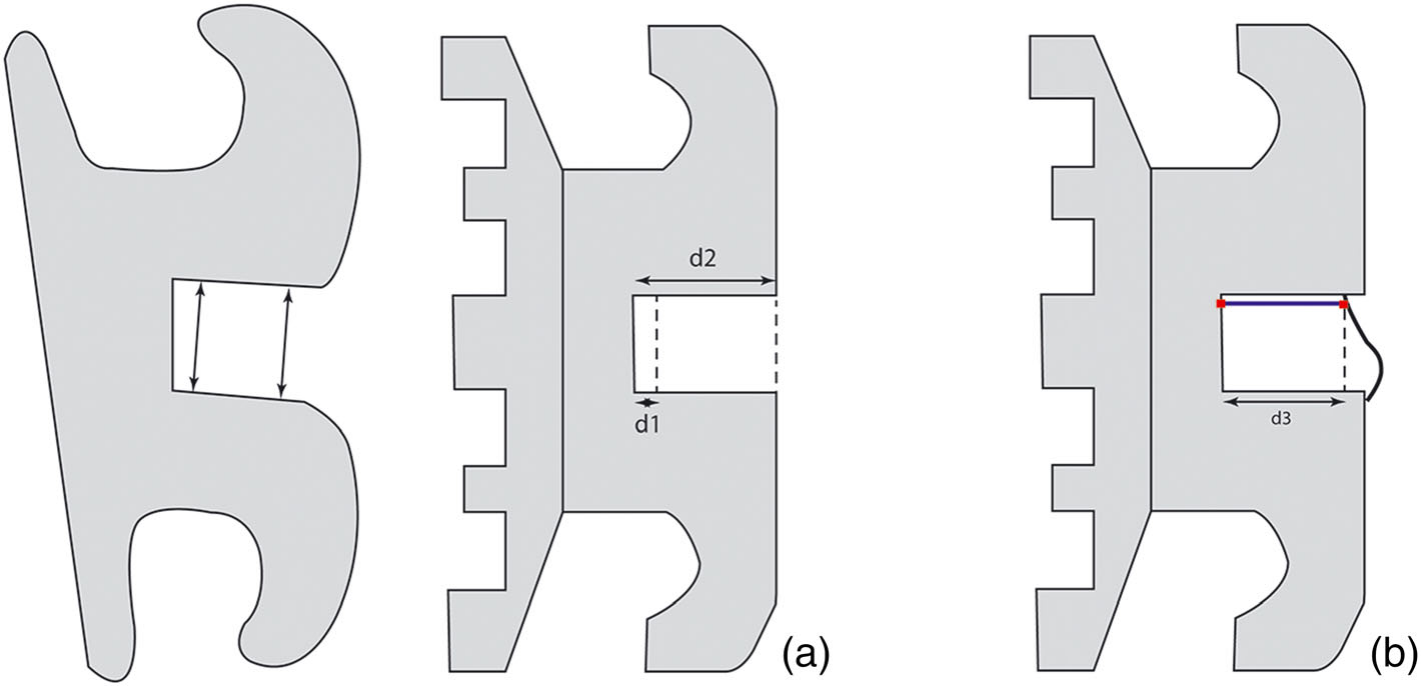
(a) Side view of a bracket, displaying base and face width measurements (d1, distance between slot base and base measurement; d2, distance between slot base and face measurement); (b) Side view of a self‐ligating bracket (d3, slot length measurement)

Each bracket slot was measured at base and at face, using ImageJ software (National Institute of Health). To prevent the bias due to the roundness of the slot angles, the measurement was done at a distance of 100 μm from the slot base. For the same reasons, the faces were measured at 610 μm (Figure 1a). In cases where the slot was not long enough, the dimensions were taken as far as possible from the base.

Slot length was evaluated for the self‐ligating brackets. This length was measured as the shortest distance between the slot base and the clip (Figure 1b).

All measurements were rounded off at to the nearest micron.

Width and length sizes were compared with those announced by the manufacturers. Bracket symmetry, slot walls parallelism, and intraseries reproducibility were also analyzed.

Wilcoxon signed‐rank test and sign test were used to compare matched samples or repeated measurements on a single sample. Data were analyzed using two‐ and three‐way ANOVA and Tukey's tests (*p* < .05).

A hundred bracket slot images were analyzed independently and randomly by two blinded examiners. The intraobserver and interobserver variability was assessed using the intraclass correlation coefficient. An intraclass correlation coefficient score close to 1 states that the correlation is strong.

Statistical analyses were performed using SPSS® v. 25.0 software (IBM Corporation, Armonk, NY). A *p* value <.05 was considered as statistically significant.

## 3 RESULTS

The comparison between slot widths and nominal values exhibited that 90% of tested brackets showed a statistically significant difference with the announced size at base, whereas 97% of the brackets were significantly different at face. The interobserver agreement was 0.96 and the intraobserver variability was 0.99 and 0.98.

Base and face widths are presented in Tables 1 and 2. At base, 11% (mesial value) to 12% (distal value) of bracket slots were significantly narrower than expected. On the opposite, 78% (distal value) to 79% (mesial value) of the slot measurements at base were significantly wider. At face, no bracket slot was significantly narrower than expected, whereas 97% were significantly wider than expected.

**Table 1:** Base slot width (mean ± SD) and average difference from nominal value (%)

Manufacturer	Conventional brackets				Self‐ligating brackets			
	18 × 25		22 × 28		18 × 25		22 × 28	
	Mean ± SD	Average difference from nominal value (%)	Mean ± SD	Average difference from nominal value (%)	Mean ± SD	Average difference from nominal value (%)	Mean ± SD	Average difference from nominal value (%)
3M	459.5 ± 30.7	+0.5	‐	‐	472.2 ± 8.3	+3.3	580.4 ± 5.2	+3.9
AO	‐	‐	542.4 ± 7.6	‐2.9	‐	‐	572.8 ± 12.4	+2.5
Dentaurum	‐	‐	579.4 ± 12.6	+3.7	‐	‐	613.1 ± 10.3	+9.7
GAC	479.9 ± 6.8	+5.0	583.0 ± 10.8	+4.3	467.9 ± 4.6	+2.3	562.5 ± 7.6	+0.7
GC	475.8 ± 6.7	+4.7	577.1 ± 15.5	+3.3	468.2 ± 4.6	+2.4	561.4 ± 5.3	+0.5
Ormco	453.6 ± 10.8	−0.8	532.2 ± 12.2	+4.8	‐	‐	567.0 ± 9.3	+1.5
RMO	481.9 ± 10.1	+5.4	586.8 ± 8.3	+5.0	‐	‐	‐	‐

**Table 2:** Face slot width (mean ± SD) and average difference from nominal value (%)

Manufacturer	Conventional brackets				Self‐ligating brackets			
	18 × 25		22 × 28		18 × 25		22 × 28	
	Mean ± SD	Average difference from nominal value (%)	Mean ± SD	Average difference from nominal value (%)	Mean ± SD	Average difference from nominal value (%)	Mean ± SD	Average difference from nominal value (%)
3M	491.2 ± 9.1	+7.4	‐	‐	483.5 ± 11.6	+5.8	592.4 ± 6.0	+6.0
AO	‐	‐	564.6 ± 12.7	+1.0	‐	‐	575.8 ± 13.0	+3.1
Dentaurum	‐	‐	592.4 ± 15.6	+6.0	‐	‐	615.6 ± 10.2	+10.2
GAC	487.0 ± 13.1	+6.5	589.3 ± 9.9	+5.5	473.8 ± 4.3	+3.6	569.8 ± 5.6	+2.0
GC	488.4 ± 7.0	+6.8	587.0 ± 10.2	+5.0	474.8 ± 5.1	+3.8	567.5 ± 6.4	+1.6
Ormco	473.2 ± 5.6	+3.5	567.1 ± 5.4	+1.5	‐	‐	572.7 ± 6.1	+2.5
RMO	509.3 ± 17.2	+11.4	614.9 ± 20.9	+10.0	‐	‐	‐	‐

There was a statistically significant three‐way interaction between manufacturer, ligating type, and material for base size (*F* = 8.365, *p* = .0001) and for face size (*F* = 2.481, *p* = .030). The manufacturer had a significant effect for base and face widths (*p* = .0001). Two‐way ANOVA did not reveal an interaction between ligating type and material for base (*F* = 3.060, *p* = .080) and for face (*F* = 0.328, *p* = .567).

Slot walls parallelism was evaluated by comparing the base and the face values for mesial and distal sides. At mesial side, 88% of the brackets showed a statistically significant difference between the base and the face (86% for conventional brackets versus 91% of the self‐ligating ones). At distal side, 85% of the brackets showed a statistically significant difference between the base and the face (88% for conventional brackets and 78% for self‐ligating ones). Significant convergence or divergence of the slot walls was also assessed, and it was shown that 1% (distal) to 3% (mesial value) of the walls were significantly convergent, whereas 84% (distal) to 85% (mesial value) were significantly divergent. No conventional bracket showed a convergence according to the mesial side measure, and no self‐ligating bracket showed a convergence according to the distal side measure.

The comparison between mesial and distal sides was made in order to evaluate bracket symmetry. It was shown that 45% of the bracket slots were asymmetrical at their bases (36% for conventional brackets and 65% for self‐ligating ones), against 34% at their faces (32% for conventional brackets and 39% for self‐ligating ones).

Slot length analysis was performed for active and passive self‐ligating brackets (Figure 1b, Table 3). The mean value for each series varied from 426.5 ± 38.2 to 737.8 ± 24.5 μm for 0.018 × 0.025‐in. brackets, and from 348.7 ± 16.0 to 701.4 ± 15.8 μm for 0.022 × 0.028‐in. brackets. Regarding the brackets with an active clip, it was observed that the mean length ranged from 348.7 ± 16.0 to 547.3 ± 33.4 μm. There was a statistically significant three‐way interaction between manufacturer, ligating type, and material for length (*F* = 8.997, *p* = .003). The manufacturer had a significant effect for length (*p* = .0001). Two‐way ANOVA did not reveal an interaction between bracket type and material (*F* = 1.439, *p* = .231).

**Table 3:** Slot length for self‐ligating brackets

Manufacturer	18 × 25		22 × 28	
	Metal	Ceramic	Metal	Ceramic
3M	638.9 ± 15.0	728.9 ± 22.0	489.7 ± 5.1	‐
AO	‐	‐	362.9 ± 16.8	358.3 ± 24.3
Dentaurum	‐	‐	592.9 ± 37.6	588.6 ± 29.8
GAC	503.7 ± 16.3	416.3 ± 41.3	501.1 ± 25.0	414.8 ± 31.0
GC	508.6 ± 14.4	411.4 ± 32.6	517.2 ± 24.0	459.0 ± 24.3
Ormco	‐	‐	704.6 ± 18.7	627.2 ± 15.7
RMO	‐	‐	‐	‐

## 4 DISCUSSION

In the present study, the purpose was to select a bracket series from the entire product range of seven different manufacturers, in order to evaluate brackets with different types of ligation mode, size, and material. Results regarding this particular parameter bring out the fact that the dimensional reproducibility is very uneven within the different series, hence preventing the practitioner from relying on the repeatability of treatment outcomes.

This study highlights the dimensional inaccuracy of a large proportion of the tested bracket series. It was shown that 90% to 97% of slot width measurements differed with stated value. Those measurements generally tended toward an enlargement of the slot and a divergence of slot walls.

Bracket dimensional variations have already been a concern for several authors whose findings were in accordance with those stated in the present study. Siatkowski described slot enlargement in 1999 and then explained such variation by an error of conversion between the American imperial tooling system and the European metric tooling, causing an oversize of brackets by 4.22% (Siatkowski, 1999). Most recent studies are more inclined to attribute these inaccuracies to imprecise manufacturing processes.

Cash et al. gathered a sample of 55 brackets of 0.022 × 0.028 in. stated dimensions, marketed by six different companies (Cash et al., 2004). They pointed out that all bracket slots were enlarged, with a maximum oversizing of +24% (Discovery Roth, Forestadent, Pforzheim, Germany). They noted that some of the slots were convergent, and some, divergent.

Arreghini et al. evaluated the dimensions of eight different brackets from three manufacturers and exhibited that all brackets tested featured oversized slots, with a maximum of +11.2% (Victory, 3M Unitek) (Arreghini et al., 2014).

Tangri et al. analyzed twenty 0.022 × 0.028‐in. brackets from five different manufacturers, using a stereomicroscope (Tangri & Kumar, 2012). A significant difference of the slot size was observed for all of the brackets, both at base and at face.

Brown et al. measured the slot dimensions of 10 bracket series approximating five sets of brackets each and reported that about 30% of the brackets were narrower than expected, whereas 15% to 20% were at least 0.001 in. larger than the nominal value (Brown, Wagner, & Choi, 2015).

Lee et al. selected seven types of 0.018 and 0.022‐in. ceramic self‐ligating brackets and observed that the slot dimensions of the different bracket systems significantly differed from each other (Lee, Lee, & Kim, 2016). All brackets presented a divergent profile with a slot face wider than the given value in all types of brackets. The greatest error was observed for the Clarity‐SL brackets (3M Unitek, Monrovia, California) with an oversizing of 30.9% of the slot face.

Focusing on interseries discrepancies, we can emphasize the significance of the irregularities between different bracket series. Indeed, if using Inspire Ice bracket series (0.022 × 0.0028 in., Ormco), slot base would be 522.0 ± 8.9 μm wide, whereas if using Dinamique C bracket series (0.022 × 0.028 in., Dentaurum), slot base would be 621.4 ± 7.8 μm wide, meaning that there is a difference of 99.4 μm between both slot widths at base, that is, 0.004 in. Furthermore, we notice that Fli Twin bracket series (0.022 × 0.028 in., RMO) presents with the most extreme enlargement at face with an increase of 96.1 μm compared with stated value. This means that bracket slot width will measure 0.026 in. at face, instead of stated 0.022 in.

Measures have already been taken to counter dimensional irregularities, with an interval of 40 μm tolerated by the Deutsches Institut für Normung (Deutsches Institut für Normung e.V, 2000). In spite of those regulations, 3% to 18% of the width of tested bracket were under the lower limit established by DIN 13971‐2 and 1% to 18% were over the upper limit (Lefebvre, Saadaoui, Olive, Renaudin, & Jordana, 2019).

Analysis of slot wall parallelism indicates that only three (Damon Q, 0.022 × 0.028 in., Ormco; Smartclip, 0.018 × 0.025 in., 3M Unitek; Ovation C, 0.022 × 0.028 in, Dentsply Gac) out of the 73 bracket series showed a convergent value on one of their side, whereas 84% to 85% of the studied brackets presented with a significant divergence. Divergence could go as up to a difference of 66.9 μm between base and face (Clarity Advanced, 0.018 × 0.025, 3M Unitek, distal measure), meaning that slot size went from 0.018 in. at base to 0.021 in. at face.

Regarding bracket asymmetry, we notice that about a third to a half of the slots were asymmetrical. Such difference between mesial and distal sides would cause a poor control of tooth tipping, especially for brackets comprising a large mesio‐distal width.

Measurement of slot length was performed for all self‐ligating bracket series, with passive or active clip. Active clip in self‐ligating brackets are expected to exert a pressure on the wire, and thus improving the insertion of the wire in the slot. Present outcomes reveal that mean slot length was highly uneven among the different active self‐ligating bracket series, the lowest being 0.014 in (Cosmetic Empower, AO), and the higher 0.023 in. (Dinamique M, Dentaurum). This will indicate that, for some bracket series, the clip will have an action as soon as the early stages of tooth leveling, whereas for others, the practitioner will have to wait for the use of a rectangular archwire with a cross‐section superior to 0.023 in. The usefulness of those active clips was already questioned by Brauchli et al. in 2011, as they stated that the spring strength was limited to 1 Nmm, which would be insufficient to produce a clinical action (Brauchli et al., 2011).

Besides the fact that dimensions are not accurate, it is relevant to note that slot inclination angles are not reliable either (Lefebvre, Olive, Saadaoui, Renaudin, & Jordana, 2018).

According to our results, we argue that, to comply with dimension regulations, improvement is required to manufacturers. Practitioners cannot fully trust the precision of the appliances available on the market and must be aware that adjustments are needed in the finishing stages of the treatment.

## 5 CONCLUSIONS

A total of 730 maxillary right central brackets manufactured by seven companies (Dentsply Gac, American Orthodontics, Rocky Mountain Orthodontics, GC Orthodontics, 3M Unitek, and Dentaurum) were studied. A proportion of 90% to 97% of the brackets evaluated displayed a statistically significant inaccuracy with the nominal value in regards to slot width. Slots were mostly oversized, with divergent walls. Manufacturer had a significant effect for base and face widths (*p* = .0001) and for length (*p* = .003).

Clinically, such variations will increase the wire‐slot play, which induces a loss of torque control. Those findings indicate that, even when using a straight wire technique, the orthodontist cannot rely only on the information incorporated into the brackets and is likely to add correction bends.

### CONFLICT OF INTERESTS

The authors of this manuscript declare that they have no conflict of interest.
